# A qualitative study of cardiovascular disease risk communication in NHS Health Check using different risk calculators: protocol for the RIsk COmmunication in NHS Health Check (RICO) study

**DOI:** 10.1186/s12875-018-0897-0

**Published:** 2019-01-14

**Authors:** Christopher J. Gidlow, Naomi J. Ellis, Lisa Cowap, Victoria Riley, Diane Crone, Elizabeth Cottrell, Sarah Grogan, Ruth Chambers, David Clark-Carter

**Affiliations:** 1 Staffordshire University, Brindley Building, Leek Road, Stoke-on-Trent, ST4 2DF UK; 2 Cardiff Metropolitan University, Cyncoed Campus, Cyncoed Road, Cardiff, CF23 6XD UK; 3 Keele University, Keele, Newcastle-under-Lyme ST5 5BG UK; 4 Manchester Metropolitan University, Manchester Campus, Bonsall Street, Manchester, M15 6GX UK; 5 Stoke-on-Trent Clinical Commissioning Group, Smithfield One Building, Stoke-on-Trent, ST1 4FA UK; 6 Staffordshire University, The Science Centre, Leek Road, Stoke-on-Trent, ST4 2DF UK

**Keywords:** Cardiovascular disease, Risk communication, Health check, Chronic disease prevention, Protection motivation theory

## Abstract

**Background:**

NHS Health Check is a national cardiovascular disease (CVD) risk assessment programme for 40–74 year olds in England, in which practitioners should assess and communicate CVD risk, supported by appropriate risk-management advice and goal-setting. This requires effective communication, to equip patients with knowledge and intention to act. Currently, the QRISK®2 10-year CVD risk score is most common way in which CVD risk is estimated. Newer tools, such as JBS3, allow manipulation of risk factors and can demonstrate the impact of positive actions. However, the use, and relative value, of these tools within CVD risk communication is unknown. We will explore practitioner and patient CVD risk perceptions when using QRISK®2 or JBS3, the associated advice or treatment offered by the practitioner, and patients’ responses.

**Methods:**

RIsk COmmunication in NHS Health Check (RICO) is a qualitative study with quantitative process evaluation. Twelve general practices in the West Midlands of England will be randomised to one of two groups: usual practice, in which practitioners use QRISK®2 to assess and communicate CVD risk; intervention, in which practitioners use JBS3. Twenty Health Checks per practice will be video-recorded (*n* = 240, 120 per group), with patients stratified by age, gender and ethnicity. Post-Health Check, video-stimulated recall (VSR) interviews will be conducted with 48 patients (n = 24 per group) and all practitioners (*n* = 12–18), using video excerpts to enhance participant recall/reflection. Patient medical record reviews will detect health-protective actions in the first 12-weeks following a Health Check (e.g., lifestyle referrals, statin prescription). Risk communication, patient response and intentions for health-protective behaviours in each group will be explored through thematic analysis of video-recorded Health Checks (using Protection Motivation Theory as a framework) and VSR interviews. Process evaluation will include between-group comparisons of quantitatively coded Health Check content and post-Health Check patient outcomes. Finally, 10 patients with the most positive intentions or behaviours will be selected for case study analysis (using all data sources).

**Discussion:**

This study will produce novel insights about the utility of QRISK®2 and JBS3 to promote patient and practitioner understanding and perception of CVD risk and associated implications for patient intentions with respect to health-protective behaviours (and underlying mechanisms). Recommendations for practice will be developed.

**Trial registration:**

ISRCTN ISRCTN10443908. Registered 7th February 2017.

**Electronic supplementary material:**

The online version of this article (10.1186/s12875-018-0897-0) contains supplementary material, which is available to authorized users.

## Background

### Cardiovascular risk communication in NHS Health Check

Cardiovascular disease (CVD) is the UK’s leading cause of mortality, accounting for 27% of all deaths [1]. The National Health Service (NHS) Health Check [2] is a strategically important national CVD risk assessment programme for adults in England aged 40–74 without certain cardiovascular-related diseases. Initiated in 2009, NHS Health Checks represent a considerable public investment. However, use of general health checks to reduce population CVD or CVD risk is much debated [3–8]. In addition to a relative dearth of evidence to support the longer-term clinical value of general health checks, or specifically relating to NHS Health Checks, little is known about the nature of Health Check consultations. Consultations should involve a practitioner (usually a Practice Nurse (PN) or Health Care Assistant (HCA)) assessing and then communicating the patient’s CVD risk to them, supported by appropriate advice and goal setting. This may range from basic lifestyle advice to referrals to the GP for medication or to relevant services (e.g., smoking cessation; dietetic). However, insights regarding exactly what happens during Health Checks are limited to retrospective qualitative data [9].

Practitioner-patient interactions are complex [10] and communicating risk is challenging [11]. For Health Checks to promote health-protective behaviours that reduce CVD risk, practitioners need to understand the risk information and be able to communicate it effectively such that patients leave the consultation with the knowledge and intention to act. A review of 70 risk-scoring methods concluded that there is no single ‘correct’ approach, but that this will depend on individual patient’s preferences and understanding, which, in turn, may differ with education status, numeracy, and personality traits, such as optimism [12]. The patients’ emotional response to the communication of risk, how and by whom the information is conveyed, presentation of risk and the influence on health behaviour, differ greatly between patients [13–16]. Poor communication of risk can cause patients anxiety and reduce confidence in health professionals [17], or may result in the perception that action is futile, but if delivered effectively, it can enhance knowledge and decision making about treatment, and can empower and create autonomy [18].

To date, there is insufficient evidence to know the nature and adequacy of CVD risk communication in NHS Health Checks. The standard CVD risk score for use in Health Checks is QRISK®2, a percentage risk of a CVD event in the next 10 years, which is integrated within in general practice medical record software. QRISK®2 has two main limitations. First, the score depends heavily on age and gender (underestimating risk in younger adults/women) and cannot account for risk from other diseases as effectively as long-term estimates [19]. Second, retrospective interview data show limited practitioner/patient understanding of percentage CVD risk [9, 20, 21], that practitioners find it difficult to explain percentage CVD risk [14, 22–24] and, in turn, patients may be unable to recall being provided with a risk score or find it confusing [9]. Further, representing percentage risk over the next 10 years (absolute risk) can be falsely reassuring [25, 26]. This is particularly problematic for individuals with low-to-moderate CVD risk who have a number of modifiable risk factors, such as smoking, obesity and hypertension [27]. These limitations have sparked interest in alternative metrics, such as heart age [3, 28–30] and lifetime risk [19], and use of multiple visual displays to present them [11].

JBS3 was launched in 2014 with a primary focus on lifetime risk [19]. It uses various visual displays (e.g., graphs of risk trajectory across life course; smiley face (‘Cates’) plots to illustrate percentage risk) and other metrics, such as Heart Age, and allows practitioners to manipulate and thus show the effects on lifetime risk trajectory of risk factor modification (e.g., smoking cessation) [19]. The potential advantages of JBS3 over QRISK®2 include: (i) measurement of lifetime risk, which is less dependent on age and gender; (ii) lifetime risk takes into account both risk from CVD and competing diseases; (iii) multiple ways in which risk information is presented could accommodate the needs and preferences of a range of patients and facilitate practitioner communication [11, 28]; (iv) ability to manipulate risk factors to demonstrate the effects of risk factor modification, which could facilitate discussion about lifestyle change or interventions; (v) heart age combines absolute risk and relative CVD risk in a way that easier to understand than percentage CVD risk [3].

In summary, we lack understanding of how risk is communicated by practitioners, and understood and used by patients in NHS Health Checks, but we do recognise limitations of percentage risk scores, such as those presented by QRISK®2 [20, 21]. Further, we can see the potential advantages of conveying risk information using more flexible and interactive platforms such as JBS3. Unless these potential advantages are evidenced to support these more adaptable and comprehensive risk communication platform, implementation of JBS3 through incorporation into general practice software systems, is unlikely.

### Aims and objectives

RIsk COmmunication in NHS Health Check (RICO) is a qualitative study and quantitative process evaluation that aims to explore practitioner and patient perceptions and understanding of CVD risk when using the JBS3 lifetime risk calculator or the QRISK®2 10-year risk calculator, the associated advice or treatment offered by the practitioner and the response of the patient. Specific study objectives are to:

1. Explore how practitioners use QRISK®2 and JBS3 to communicate CVD risk in the consultation
2. Explore how patients respond to the risk information
3. Explore how QRISK®2 and JBS3 promote patient and practitioner understanding and perception of CVD risk
4. Explore patient intentions with respect to health-protective behaviours
5. Explore mechanisms by which intentions for health-protective behaviours are elicited
6. Make recommendations regarding use of QRISK®2 or JBS3 in Health Checks.

### Theoretical basis

**Fig. 1:**
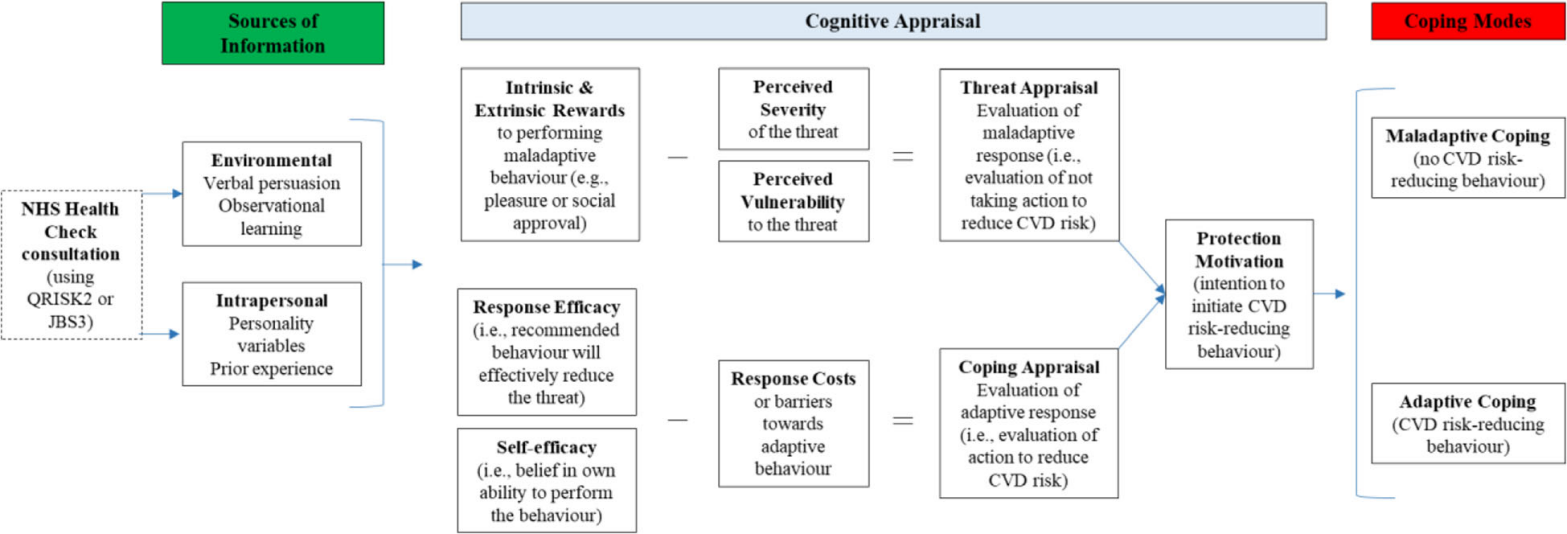
Protection Motivation Theory model adapted to proposed study context (adapted from [33, 35])

Given the complexity of practitioner-patient interactions [31, 32] and the translation of risk information into health-protective behaviour [33], to ensure a comprehensive enquiry about the relative values of JBS3 and QRISK2, we have used a theoretical framework based on the revised Protection Motivation Theory (PMT) [34]. Within the PMT, ‘protection motivation’ refers to the intention to undertake health-protective behaviour resulting from the cognitive appraisals (or internal assessments); CVD risk communication could be a key source of information feeding into such appraisals (Fig. 1).

PMT is informed by fear-drive models, which recognise that behaviour change can be prompted by fear-inducing communications that motivate action to reduce the perceived threat (or risk) [33, 35]. However, protection motivation is influenced by two cognitive appraisals; appraisals of the threat (risk of CVD) and coping (consequences undertaking positive behaviour change). Threat appraisal evaluates maladaptive responses; i.e., not initiating positive behaviours in response to recognising an elevated CVD risk. This considers the source of the threat (i.e., practitioner/Health Check), intrinsic rewards (e.g., enjoyment associated with health risk behaviour) and extrinsic rewards (e.g., social approval), and the perception of the threat (perceived severity and personal vulnerability). Coping appraisal evaluates the adaptive response to cope with the threat (i.e., CVD risk), and considers the likelihood that positive behaviour change (adaptive response) will reduce their risk (response efficacy), their own ability to make the necessary changes (self-efficacy), and the burdens of, or barriers to, making the change (response costs) [33, 34, 36, 37]. Threat and coping appraisals are influenced by both environmental aspects (e.g., persuasive communication and observational learning) and intrapersonal variables (e.g., personality and feedback from prior experience of both positive (adaptive) and negative (maladaptive) behaviours) [33]. In the context of this study, PMT underlines the key role that practitioners have in providing information on CVD risk (vulnerability) and incorporating a patient’s beliefs, priorities and experiences into strategies to reduce this risk so that patients feel they can achieve adaptive behaviours [34] and subsequent health outcomes.

PMT is particularly pertinent to study the relative merits of different CVD risk calculators and the mechanisms by which they might promote positive behaviour change for several reasons. First, it was initially developed to examine intention to adopt behaviours relating to disease prevention [38]. Second, it does not assume rationality in behaviour choices [33, 39]; that is, people will undertake unhealthy behaviours as they serve other purposes, for example, enjoyment or social integration. Third, its components have been associated with (intention for) behaviour change in relevant contexts (e.g., smoking cessation, exercise) [34, 36] and, fourth, it provides an understanding of why attitudes and behaviour can change when people are confronted with threats (i.e., the mechanisms) [33].

## Methods/design

### Design and setting

**Fig. 2:**
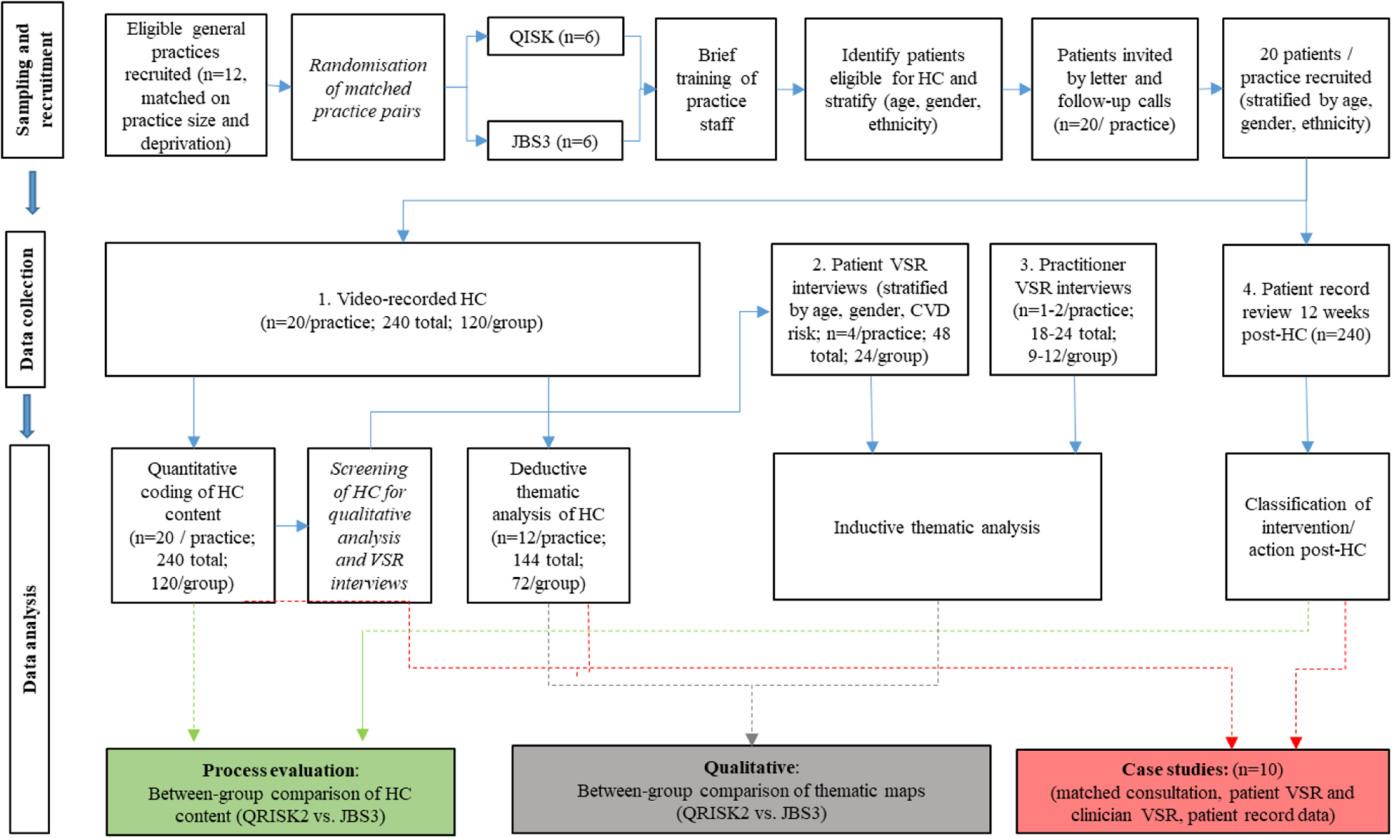
Flow diagram of study processes

This qualitative study, which includes a quantitative process evaluation, will be undertaken in 12 general practices in the West Midlands that already deliver NHS Health Checks. Six practice pairs, approximately matched on practice size and deprivation, will be randomly assigned to one of two groups: QRISK®2 (usual practice) - practitioners continue to use QRISK®2 to communicate CVD risk during Health Checks; JBS3 (intervention) - practitioners use the JBS3 CVD risk calculator following brief training about the platform, but no training will be provided about risk communication. Participating practices will video-record their NHS Health Checks using the allocated CVD risk calculator over until 20 useable consultations are recorded. As summarised in Fig. 2 and detailed below, data collection will comprise: (1) Video-recording NHS Health Check consultations; (2) Post-consultation video-stimulated recall (VSR) interviews with patients and practitioners within 2 weeks, using excerpts from recorded health checks to facilitate recall and reflection; (3) Patient medical record reviews 12-weeks post-Health Check to determine subsequent action (e.g., GP appointment, lifestyle referral, statin prescription).

### Sample

#### General practices

General practices that meet the following criteria will be recruited: a) deliver NHS Health Checks; b) already use the QRISK®2 percentage risk score in Health Checks; currently (or willing to) deliver Health Checks in specific clinics to facilitate data collection; c) are signed up to the ‘incentive scheme’ implemented by the Clinical Research Network (CRN) to ensure the GP practice is ‘research ready’; d) are willing to participate.

General practices will be stratified using data on practice list size and deprivation level of the practice location [40] to provide a proxy measure of typical socio-economic status of the practice population (Table 1).

**Table 1:** Stratified sampling of six practices per group based on deprivation and list size

	Deprivation	
Practice list size	Most deprived 50%	Least deprived 50%
Small-Medium (< 8000)	2 QRISK®2; 2 JBS3	2 QRISK®2; 2 JBS3
Large (≥8000)	1 QRISK®2; 1 JBS3	1 QRISK®2; 1 JBS3

#### Patients

The patient population will be those eligible for NHS Health Checks based on national criteria. These exclude people who: a) are outside the target age range (40–74 years); b) have existing diagnoses for certain cardiovascular-related chronic conditions; c) are taking statins; d) have had a NHS Health Check in the last 5 years; e) are known to be at high risk (≥20% 10-year CVD risk score) [41] .

#### Practitioners

Participating practitioners will be the healthcare professionals who usually deliver Health Checks in participating practices and who are willing to participate; usually one to two Practice Nurses (PN) or Health Care Assistants (HCA) per practice (*n* = 12–24).

### Recruitment

#### Practice sampling

The CRN will facilitate practice sampling. Briefly, this will involve an initial email to ‘research ready’ practices inviting expressions of interest. To identify willing and eligible practices, those expressing interest will be followed up with telephone calls and visits as appropriate. Practice participation will be incentivised through financial reimbursement of service support costs and remuneration for completing all parts of the study. Following practice-level consent, practice pairs matched on size and deprivation (Table 1), will be randomly assigned to the QRISK®2 or JBS3 group using a random number generator in MS Excel. After randomisation, the research team will undertake an initiation meeting at the practices to provide further information and basic training for staff involved.

#### Patient and practitioner sampling

There will be three levels of patient sampling.

1) Total sample (*n* = 240): To achieve the 144 recorded consultations suitable for qualitative analysis (12 per practice allowing for non-attendances and consultations with no/minimal discussion of CVD risk), Health Check clinics would be recorded until 20 recordings per practice (240 total) have been achieved. In each practice, searches of the patient database will identify the cohort of eligible patients who will be stratified according to gender, age and ethnicity to ensure representation from different demographic groups (Table 2).

**Table 2:** Stratified sampling of the 20 patients per practice to be invited for recorded Health Checks

		Gender	
		Female	Male
Age (yr)	40–54 yr	4 (3 WBRI/1 BAME)	4 (3 WBRI/1 BAME)
	55–64 yr	3 (2 WBRI/1 BAME)	3 (2 WBRI/1 BAME)
	65–74 yr	3 (2 WBRI/1 BAME)	3 (2 WBRI/1 BAME)

WBRI, White British; BAME, Black, Asian, Minority Ethnic

2) Qualitative analysis (*n* = 144): Video recordings will be screened within 48 h of filming. This will involve quantitative coding of the content of the consultation to identify those suitable for qualitative analysis (12 per practice) and VSR interview (4 per practice). Where risk is not discussed by patient or practitioner, the patient’s data would not be used for either.

3) VSR interviews (*n* = 48): VSR interviews will be conducted with 48 patients (24 per group) sampled from the 144 recorded Health Checks, stratified by gender, age and CVD risk (Table 3).

**Table 3:** Example of stratified sampling of VSR patient interviews per group based on age, CVD risk and gender

		CVD Risk^a^	
		Low (<10%)	Medium-High (≥10%)
Age (yr)	40–54 yr	2 m / 2 f	2 m / 2 f
	55–64 yr	2 m / 2 f	2 m / 2 f
	65–74 yr	2 m / 2 f	2 m / 2 f

^a^QRISK percentage 10-year risk would be used for stratification purposes for consistency across both groups

The proposed total of 144 recorded consultations (12 per practice) with 48 patient VSR interviews and 18 practitioner VSR interviews, is comparable with other studies using audio-recording of similar consultations to explore CVD risk communication in patients with psoriasis (*n* = 130 in 10 practices [42]) and the number of interviews in VSR studies (*n* = 9–39 [43]).

All practitioners who will deliver the video-recorded Health Check clinics will be asked to participate in VSR interviews.

### Groups

*QRISK®2 group* (usual practice): Practitioners will deliver Health Checks as usual, using the QRISK®2 risk calculator as per usual practice.

*JBS3 group* (intervention): Practitioners will deliver Health Checks using the JBS3 risk calculator. An introductory session with practitioners will establish the requirements to: avoid using QRISK®2 to communicate CVD risk; use the first two ‘output’ screens as a minimum (Heart Age and Survival Age); show the effects of intervention through modifying risk scores (e.g., lowering blood pressure, smoking cessation); practice with JBS3 in at least two Health Checks prior to video-recorded clinics.

### Data collection procedures

#### Video-recorded health checks

Digital camcorders will be positioned in the Health Check clinic rooms to provide an audio-visual record of consultations. Informed by Patient Public Involvement (PPI) and pilot work, cameras will be positioned to capture both patient and practitioner, but prioritising the view of the patient. Video recordings will be screened (during quantitative coding). If there is no discussion of CVD risk, this will be noted, and the file retained. For consultations that involve discussion of CVD risk, the audio-record will be separated from the visual (using Adobe Premiere Pro) for transcription and qualitative analysis (*n* = 12 per practice; 144 total).

#### Semi-structured VSR interviews with patients and practitioners

Semi-structured one-to-one VSR interviews with patients will be arranged within the 2 weeks following their Health Check; for practitioners, VSR interviews will be within 2 weeks of their final recorded Health Check. After each clinic, recorded Heath Checks will be watched to identify sections of the consultation to use in VSR interviews that relate to discussion of the CVD risk score, modification of the risk score, and practitioner advice, recommendations and interventions. For practitioner VSR interviews, video excerpts will be taken from the consultations with patients also selected for VSR interviews. The semi-structured VSR interviews will follow a pre-piloted process and topic guide (Additional file 1: Table S1), with slight variation depending on whether the patient/practitioner are in the QRISK®2 or JBS3 group. All VSR interviews will be audio-recorded and transcribed verbatim for analysis.

#### Patient medical record review

Data from the 12 weeks following the Health Check will be extracted from patient medical records to identify any subsequent activity. This will be used to identify any subsequent recorded actions or interventions (e.g., GP appointment, lifestyle referral, or statin prescription).

### Patient and public involvement

Patient and Public Involvement (PPI) activities informed study development and will continue to its completion. Three PPI strategies have been used. First, we have engaged with Patient Participation Groups (PPG) by attending PPG meetings at three general practices on two occasions to gather opinion on the study concept and overall design, and subsequently, the methods and protocols. One PPG facilitated the completion of four mock Health Checks (with the Practice Nurse and four PPG members) to allow testing of protocols including camera placement, video-recording quality, participant consent and debrief processes, development of the quantitative and qualitative coding frameworks, post-processing of video for VSR excerpts and development of the VSR topic guide and protocols. Second, two patient representatives sit on the Study Steering Committee for ongoing involvement of patients in project management. Third, a virtual study patient group has been established using a closed Facebook group. This  has allowed engagement with many patients and public (current membership ~ 295) who have provided rapid feedback on a range of issues (e.g., consent forms, participant information sheets, camera placement).

### Data analysis

Qualitative and quantitative data will be analysed to inform the quantitative process evaluation, qualitative outcomes and case studies (Fig. 2). The processes are summarised by data source.

#### Qualitative data - recorded health check consultations

Qualitative data will be analysed using thematic analysis, following the six stage process described by Braun and Clarke [44] (Table 4). Health Check consultation data will be analysed deductively. A coding template will be developed based around the PMT (Fig. 1). Each consultation video and associated transcript will be uploaded to NVivo for analysis, using the visual information from the videos for additional context (see Additional file 2: Table S2 which gives examples of  how behaviours can be used to determine level of engagement). Analysis will be completed separately for consultations in the QRISK®2 and JBS3 groups for comparison. This will allow interpretation of how QRISK®2 and JBS3 are used to communicate risk in the context of PMT factors (e.g., verbal persuasion, influencing patient prior beliefs and priorities; Obj.1) and how patients respond (Obj.2), which will reflect the nature of their appraisal (threat/coping) within the consultation. Both will allow inferences about the mechanisms at work in consultations that appear more/less successful (Obj. 5).

**Table 4:** Process of Thematic Analysis (adapted from [45])

Phase		Summary
Phase 1	Familiarisation	Analysis will start with a period of familiarisation involving watching and re-watching the video-recorded consultation (or listening to audio-records in the cases of interviews), noting initial thoughts in the transcript
Phase 2	Initial coding	For deductive analysis, codes from the PMT template will be applied to the transcript independently by two researchers; for inductive analysis, codes will be generated based on interesting features, and recurrent patterns, in the data. For both inductive and deductive analysis, the researchers will then go back through and check their own codes, before discussion to verify and agree final codes.
Phase 3	Searching for themes	Agreed codes will be collated into potential themes, gathering all data relevant to each potential theme.
Phase 4	Reviewing themes	Constant comparison will be used to check themes by revisiting data to ensure they are representative, and then generating a thematic ‘map’ of the analysis.
Phase 5	Defining and naming themes	Ongoing analysis to refine the specifics of each theme, and the overall story, generating clear definitions and names for each theme
Phase 6	Reporting	Illustrative extracts will be selected to include in a narrative that tells the overall story.

#### Qualitative data - semi-structured VSR interviews with patients and practitioners

Patient VSR interview transcripts will be analysed using inductive thematic analysis, where codes and themes are generated from data based on individual reflections, perceptions and experiences (Table 4). This will be completed separately for QRISK®2 and JBS3 groups for comparison. The resulting thematic map for each group will provide insight into patient perceptions and understanding of CVD risk (Obj. 3), with video-stimulated reflections on that experience, and further reflections on their thoughts, feelings and intentions to undertake health-protective behaviour following the Health Check (Obj. 4). Data will also allow inferences about the underlying mechanisms (Obj. 5).

Similarly, inductive thematic analysis will be used to analyse practitioner VSR interview transcripts, separately for QRISK®2 and JBS3 groups. The resulting thematic map for each group will provide insight into their perceptions and understanding of CVD risk (Obj. 3), with video-stimulated reflections on aspects such as how they communicate risk in consultations, their use of the calculator, the types of advice they offer, patient responses (Obj. 4), allowing inferences about the underlying mechanisms (Obj. 5).

#### Quantitative - content of health check consultations

The content of the recorded consultations will be characterised using a coding framework that involves second-by-second coding of Health Check content. The framework comprises 36 items grouped in to six categories (patient-practitioner communication, general Health Check processes, risk dialogue, CVD risk factors, lifestyle interventions, medical interventions). The resulting data will provide aggregate indicators for each consultation to allow between-group comparisons (e.g., proportion of Health Check considered practitioner- or patient-dominated; proportion of time discussing risk; proportion of time discussing intervention/changes; number of times the practitioner manipulated the risk score to illustrate amenability of risk to change).

The development of the coding process and guide was iterative. Four “mock” Health Checks were undertaken by practitioners and PPI volunteers. These were video-recorded and two researchers (LC; NE) coded the consultations by consensus to reach consistency in approach. A third researcher (VR) then coded all four consultations independently. Intraclass Correlation Coefficients (ICCs) were calculated and demonstrated excellent inter-rater reliability (ICCs ranged from .968 to .995). The resulting framework will be refined during a training/checking phase with study data, whereby an additional four recorded Health Checks (2% of total) will again be independently coded by two researchers and inter-rater agreement assessed. Once finalised, two researchers (LC; VR) will code the remaining Health Check recordings independently (118 each). For every 20 coded consultations (8% of total), two would be subject to independent verification (independent coding and calculation of ICCs). This will mitigate the risk of coder drift throughout the study and provide independent verification of 10% of consultations overall.

A between subjects t-test or non-parametric equivalent will be used for between-group comparisons of key outcomes for Health Check content (e.g., proportion of time spent discussing CVD risk). To explore possible cohort effects within the data, ICCs will be calculated (i.e., to examine possible clustering within practices). Multi-level modelling is not appropriate; the study is designed to allow for novel qualitative enquiry and is not powered for multi-level statistical analysis.

#### Quantitative – Patient medical record review

Data from patient medical records will be tabulated for an exploratory descriptive comparison of the two groups. The primary purpose will be to provide additional context to qualitative data, particularly the VSR interview and case study analysis (see below). Between-group comparisons will be explored as above.

#### Within-case analysis

A subsample of 10 patients who demonstrate the most positive intentions and/or behaviours to reduce CVD risk following the Health Check will be selected for case study analysis, drawing on all data for each patient. The aim is to further explore apparent mechanisms by which the risk calculators may lead to changes in patient or practitioner behaviour (Obj. 5). A coding framework for deductive analysis of qualitative data based on potential mechanisms of eliciting health-protective intentions/behaviours will be generated from findings in recorded Health Checks, and VSR patient and practitioner interviews, and applied to qualitative data in each case study (Health Check, and patient and practitioner VSR interviews). The quantitative data on Health Check content and subsequent actions would be used to provide a basic profile for each patient to aid interpretation.

### Sample size

A priori determination of sample sizes for qualitative research is a point of contention [45, 46]. For the present study, it was necessary to estimate requirements for the patient VSR interviews and use this to inform the total number of recorded Health Checks required per practice. As summarised in Table 3, 48 VSR patient interviews (24 per group, 4 per practice) will allow patient sampling stratified by gender, age and CVD risk, and provide a sample size that compares favourably with studies using VSR or audio-recordings of primary care consultations (ranging from *n* = 9–44 [42, 43]). These 48 recorded Health Checks will be selected (with stratification) from 144 (72 per group, 12 per practice) that are subject to deductive qualitative analysis; i.e., 12 per practice was deemed sufficient to allow stratified sampling of four patients per practice. To obtain the 144 recorded Health Checks that are suitable for qualitative analysis, we will aim to record 240 (120 per group, 20 per practice). This oversampling will serve two purposes. First, it will allow for exclusions due to non-attendance, technical issues and Health Checks that contain little or no discussion of CVD risk. Second, with 120 consultations per group, using a between subjects t-test with a two-tailed probability and alpha of .05, we will have statistical power of at least .8 to detect a small to medium effect (Cohen’s d) = 0.37. It will also mean that the effect sizes derived from the study will have good levels of precision for estimating the effect sizes in future studies and so provide more accurate power analysis for such studies.

## Discussion

This innovative study is, to our knowledge, the first to examine current risk communication practice in NHS Health Check (using QRISK®2), the potential of using the JBS3 lifetime risk calculator, and to apply novel video-recording methodological approaches in this context. The multi-faceted methodological approach has many advantages. First, video-recordings will provide an objective and ‘real-time’ record for quantitative and qualitative analysis of Health Checks. Second, video-recorded Health Checks will allow analysis of both verbal and non-verbal communication, providing a comprehensive account, with the sensitivity to capture subtle details [47]. Capturing nonverbal behaviour can convey additional emotional information that is important in the study of practitioner-patient relationships [48]. Third, VSR interviews will enhance participant recall of thoughts, perceptions and emotions during the consultation, and allow a considered reflection on their related intentions and actions [31].

The outcomes will have important implications. The national NHS Health Check programme, which remains one of only three mandatory functions included in the 2012 Health and Social Care Act and has political backing as evidenced by inclusion in *Living Well for Longer: A call to action to reduce avoidable premature mortality* [49]. New insight from our data will inform recommendations for which tool should be endorsed for Health Checks and how practitioners should make best use of them. However, in a period of growing budgetary pressure, this work has value regardless of the future of NHS Health Check as the need to effectively communicate CVD risk and prompt positive behaviour change to protect against future disease will remain a key component of primary care.

## Additional files

Additional file 1: **Table S1**. Outline topic guides for Video-stimulated recall (VSR) interviews (DOCX 16 kb)

Additional file 2: **Table S2**. Non-verbal behaviour coding to characterise patient-practitioner engagement (content adapted from Medical Interaction Process System (MIPS) [1], Schmid Mast et al. [2] and Henry et al. [3]) (DOCX 20 kb)

## Abbreviations

CVD: Cardiovascular disease
JBS3: Joint British Societies lifetime CVD risk calculator
NHS: National Health Service
PMT: Protection Motivation Theory
PPG: Patient Participation Group
PPI: Patient and Public Involvement
QRISK®2: Calculator to estimate the risk of having a heart attack or stroke over the 10-years
VSR: Video-stimulated recall
