# Supplementary material for: A qualitative study of cardiovascular disease risk communication in NHS Health Check using different risk calculators: protocol for the RIsk COmmunication in NHS Health Check (RICO) study
Source: BMC Fam Pract. 2019 Jan 14;20:11. doi: 10.1186/s12875-018-0897-0 (PMC6332912; doi:10.1186/s12875-018-0897-0)
Supplement: Supplementary file 1 — Table S1. Outline topic guides for Video-stimulated recall (VSR) interviews (DOCX 16 kb) [file 12875_2018_897_MOESM1_ESM.docx]

**Supplemental file S1.** Outline topic guides for Video-stimulated recall (VSR) interviews

1. Patient VSR interviews

| - Preliminary questions relating to experiences and perceptions of the Health Check - Questions relating to CVD risk score - *Participant shown video excerpt of risk score discussion* - Follow-up questions asking patients to reflect on specific aspects of the consultation (e.g., conversation around CVD risk, the terms used, the practitioner’s advice, their intentions and subsequent behaviour) - If practitioners modified the risk score to illustrate the effect of intervention (e.g., lifestyle change), participants will be asked what they remember - *Participant shown video excerpt of risk score modification* - Participants asked to reflect again on this part of the consultation - Questions relating to interventions and practitioner advice/recommendations - *Participant shown video excerpt of all recommendations made by practitioner* - Questions relating to subsequent intentions and actions - Questions relating to presentation of risk information (e.g., Heart Age, lifetime risk, visual displays, risk score modification; based on experience in JBS3 group and hypothetical in QRISK®2). - Questions regarding overall experience of Health Check. |
| --- |

1. Practitioner VSR interviews

| **QRISK®2:**   1. Preliminary questions relating to experiences and perceptions of delivering Health Checks 2. Questions relating to CVD risk communication and risk scores  - *Shown video excerpt of QRISK®2 % risk score discussion*  1. Follow-up questions asking practitioner to reflect on how they approach this  - *Shown video excerpt of QRISK®2 % risk score discussion focusing on patient reaction*  1. Follow-up questions asking practitioner to reflect on patient response  - *Shown video excerpt of risk score modification (if it happened)*  1. If practitioners modified the risk score to illustrate the effect of intervention, asked about perceived purpose and impact 2. Questions relating to alternative approaches to presenting CVD risk information (e.g., Heart Age, lifetime risk, visual displays, risk score modification) 3. If risk scores not modified, ask hypothetical questions regarding usefulness and impact 4. Questions relating to interventions and advice/recommendations  - *Shown video excerpt of all recommendations*  1. Questions relating to interventions and advice/recommendations 2. Questions regarding overall experience of delivering Health Checks and areas for improvement. | **JBS3:**   1. Same 2. Same  - *Shown video excerpts of JBS3 % risk score, Heart Age, Survival Age discussions*  1. Follow-up questions asking practitioner to reflect on how they approach this  - *Shown video excerpts of JBS3 % risk score, Heart Age, Survival Age discussions focusing on patient reaction*  1. Same  - *Shown video excerpt of risk score modification (if it happened)*  1. Same 2. Questions relating to experiences and perceptions of JBS3 (including comparison with QRISK®2) 3. Questions relating to interventions and advice/recommendations  - *Shown video excerpt of all recommendations*  1. Questions relating to interventions and advice/recommendations 2. Questions regarding overall experience of delivering Health Checks and areas for improvement. |
| --- | --- |
|  |  |
