# Supplementary material for: A qualitative study of cardiovascular disease risk communication in NHS Health Check using different risk calculators: protocol for the RIsk COmmunication in NHS Health Check (RICO) study
Source: BMC Fam Pract. 2019 Jan 14;20:11. doi: 10.1186/s12875-018-0897-0 (PMC6332912; doi:10.1186/s12875-018-0897-0)
Supplement: Supplementary file 2 — Table S2. Non-verbal behaviour coding to characterise patient-practitioner engagement (content adapted from Medical Interaction Process System (MIPS) [1], Schmid Mast et al. [2] and Henry et al. [3]) (DOCX 20 kb) [file 12875_2018_897_MOESM2_ESM.docx]

**Supplemental file S2.** Non-verbal behaviour coding to characterise patient-practitioner engagement (content adapted from Medical Interaction Process System (MIPS) [1], Schmid Mast et al [2] and Henry et al. [3])

| **Behaviour type** | **Behaviour** | **Engaged - positive** | **Engaged - negative** | **Passive / Disengaged** |
| --- | --- | --- | --- | --- |
| **Practitioner and Patient** | |  | | |
| Proxemics | Body orientation | Towards one another | Towards one another | Twisted / away from one another |
|  | Looking at computer screen | Both engaged with screen / discussing info presented | Both engaged with screen / discussing info presented | Patient not engaging with info presented |
|  | Distance | Close | Close | Far |
| Gesture/body language | Posture | Open | Closed | Closed |
|  | Body lean | Forward | Forward or backward | Backward |
|  | Shrugging shoulders | No | Yes | Yes |
|  |  |  |  |  |
|  | Nodding | Yes | No, shaking head | No |
| Facial expression | Eye contact | Look at person / mutual gaze | Look at person / mutual gaze | Look at floor |
|  | Smiling | Yes | No | No |
| Vocal cues | Back channels (i.e., short interjections while listening, *‘uh-huh and mm-mm’*) | Yes | No | No |
|  | Questioning | Patient asks searching questions, shows interest in response | Patient asking negative short questions, dismisses or criticises / does not listen to response/ puts barriers up e.g. “how am I supposed to deal with that when XYZ are happening?” | Patient does not ask any questions |
|  | Modulation of voice | Expressive | Either | Monotonic  Dominant |
|  | Sighs | No | Patient sighs | Patient sighs |
| **Practitioner only** |  |  |  |  |
| Vocal cues | Talking while doing something else | No |  | Yes (e.g., reading, writing) |

1. Ford S, Hall A, Ratcliffe D, Fallowfield L. The Medical Interaction Process System (MIPS): an instrument for analysing interviews of oncologists and patients with cancer. Soc. Sci. Med. 2000;50:553–66.

2. Mast MS, Hall JA, Klöckner C, Choi E. Physician Gender Affects How Physician Nonverbal Behavior Is Related to Patient Satisfaction. Med. Care. 2008;46:1212–8.

3. Henry SG, Forman JH, Fetters MD. “How do you know what Aunt Martha looks like?” A video elicitation study exploring tacit clues in doctor–patient interactions. J. Eval. Clin. Pract. 2011;17:933–9. A
